# Plasmonic nanoantenna hydrophones

**DOI:** 10.1038/srep32892

**Published:** 2016-09-09

**Authors:** Ivan S. Maksymov, Andrew D. Greentree

**Affiliations:** 1 ARC Centre of Excellence for Nanoscale BioPhotonics, School of Science, RMIT University, Melbourne, VIC 3001, Australia

## Abstract

Ultrasound is a valuable biomedical imaging modality and diagnostic tool. Here we theoretically demonstrate that a single dipole plasmonic nanoantenna can be used as an optical hydrophone for MHz-range ultrasound. The nanoantenna is tuned to operate on a high-order plasmon mode, which provides an increased sensitivity to ultrasound in contrast to the usual approach of using the fundamental dipolar plasmon resonance. Plasmonic nanoantenna hydrophones may be useful for ultrasonic imaging of biological cells, cancer tissues or small blood vessels, as well as for Brillouin spectroscopy at the nanoscale.

Control of light at the nanoscale is a burgeoning field, and plasmonic nanoantennae (NA) are fast emerging as one of the most important tools for collecting, emitting and more generally controlling light with nanoscale (sub wavelength) elements^1^,^2^. This functionality derives from strong coupling of optical fields to plasmon modes in the NA, where the resonance of the plasmon modes can be controlled by the geometry of the nanoscale elements.

A hydrophone is a microphone designed to be used underwater for listening to underwater sound. In conventional hydrophones, the acoustic energy mechanically moves a transducer that in turn generates a voltage at the acoustic frequency. Optical hydrophones use frequency modulation of a light beam that Doppler shifts in response to acoustic pressure variations^3^,^4^. They have an increased sensitivity, greater discrimination against noise and improved directionality as compared with conventional hydrophones^4^.

The dimensions of state-of-the-art miniaturised optical ultrasonic hydrophones are comparable with the wavelength of ultrasound in water (∼150 *μ*m at 10 MHz)^5^,^6^,^7^,^8^. These devices benefit from the advances in integrated photonics or exploit the unique optical properties of plasmonic metamaterials, such as, for example, a macroscopic quasi-periodic array of metal nanopillars^8^.

Here we theoretically demonstrate that a single dipole plasmonic NA can be employed as an optical ultrasonic hydrophone (Fig. 1). We show that a single NA produces a considerable (∼60-fold) enhancement in sensitivity to ultrasound as compared with the case of conventional hydrophones^3^,^4^ in which light travels in bulk water.

The proposed hydrophone can be used in difficult to reach places such as blood vessels, which is important for intravascular ultrasound (IVUS) and intravascular photoacoustic (IVPA) imaging^9^. In IVPA imaging, an optical fibre is used to deliver light to a remote location inside a blood vessel. Because plasmonic NA’s can be coupled with optical fibres^10^,^11^, we envision that a single ultrasound-sensitive NA, or an array of such NA’s, can be integrated with a fibre used in IVPA imaging [Fig. 1(b)].

Other embodiments of the hydrophone may also be possible and each embodiment will introduce a peculiarity to the hydrophone operation. Thus, for simplicity, in what follows we assume that the NA is suspended in water. In our discussion, the ultrasound reception is via the detection of optical signals arising due to the Brillouin light scattering (BLS) from ultrasound^3^. The BLS effect is one of the physical mechanisms that enables optical hydrophones to sense sound^3^,^4^. Moreover, BLS spectral analysis has been proven to be a convenient tool for ultrasonic imaging of biological cells^12^ and *in-vivo* human eye imaging^13^. Consequently, we also choose to analyse BLS spectra of the NA to demonstrate the operation of our hydrophone.

The BLS effect is mediated by dynamic fluctuations in the strain field that are caused by ultrasound and lead to perturbations in the dielectric permittivity of the medium^3^. These fluctuating optical inhomogeneities result in inelastic scattering of the light as it passes through the medium. Very small amplitudes of dielectric permittivity perturbations of the medium are viewed by the incident light wave as a moving diffraction grating. Therefore the BLS effect can be explained by the two concepts of Bragg reflection and Doppler shift. As a result, the spectrum of the scattered light has the central Rayleigh peak and two Brillouin peaks shifted from the optical frequency by the frequency of the ultrasound^3^.

The intensity of Brillouin peaks is typically low because of very low amplitudes of dielectric permittivity perturbations, which requires the use of sophisticated and cumbersome optical systems^14^. Recently, it was demonstrated that the intensity of Brillouin peaks may be increases when a metal nanostructure that supports surface plasmon modes is fabricated on top of an acoustic waveguide^15^,^16^. Thus, our hydrophone may also be used to improve the sensitivity of the BLS spectroscopy technique.

Typically, the frequency of ultrasound detected with BLS spectroscopy is in the range from several MHz to several GHz (hypersound^3^). However, we consider MHz-range ultrasound because it is used in IVUS and IVPA imaging^9^. The outer diameter of combined IVUS/IVPA catheter tips is ∼1.5 mm mostly because of the large dimensions of the ultrasound transducer, which allows accessing large blood vessels only. Thus, because of its ultrasmall dimensions the proposed plasmonic NA-based optical hydrophone may be useful in novel miniature IVUS/IVPA tips suitable for imaging of small diameter blood vessels (<0.5 mm diameter).

## Optical Properties of the Nanoantenna

While there are many different NA designs^1^,^2^, our focus on a dipole NA is justified by its small metal volume, i.e. lower light absorption losses. We are interested not only in the response of the dipole NA to the fundamental dipolar resonance, but also to higher-order plasmon modes^17^, which are analogous to the modes of a Fabry-Perot resonator. Lower radiative losses associated with these modes lead to a higher electric field confinement near the surface of the NA as compared with the fundamental dipolar mode.

Different Fabry-Perot structures and other types of optical resonators have been employed for ultrasound detection because the presence of the resonator effectively increases the interaction time of light with the acoustic wave^5^,^6^,^7^. However, in such structures the intensity of light interacting with acoustic waves is low, which implies that light cannot generate new acoustic signals or cause changes in the refractive index of the medium.

Consequently, similar to Fabry-Perot-based optical hydrophones, plasmonic NA’s supporting higher-order modes may be employed to detect ultrasound by effectively increasing the interaction of light with acoustic waves. (But this increase is not expected to be significant when the NA operates at the dipolar mode that does exhibit a Fabry-Perot behaviour). However, the dimensions of plasmonic NA’s are smaller as compared with photonic Fabry-Perot structures, which makes it possible to create an ultrasmall hydrophone based on a single NA.

Hereafter, we consider a silver NA with a square cross-section *w* = 30 nm and length *L* = 340 nm. We choose silver because of its low absorption losses as compared with other metals such as, e.g., nickel^2^. However, a NA with the same dimensions but made of gold, which is another metal often used in plasmonics, will exhibit similar optical properties. Moreover, the elastic properties of the constituent metal, such as the Young’s modulus *E* and the Poisson’s ratio *v*, are also relevant to our analysis. Because *E* = 83 GPa and *v* = 0.37 of silver are close to *E* = 79 GPa and *v* = 0.42 of gold^18^, it is safe to assume that acoustic properties of the silver and gold NA’s should also be similar. Whereas *w* = 30 nm is the typical parameter for dipole plasmonic NA’s^2^, according to the wavelength scaling rule^1^ *L* = 340 nm is longer than a typical length of dipole NA’s made of noble metals. Thus, due to its increased length, the NA is designed to support higher-order plasmon resonances in the visible and near-IR spectral ranges. This design is confirmed by numerical simulations in the following section.

### 3D model of the nanoantenna

In our model, the nanoantenna is immersed in water. Water is the main component of all body fluids and soft tissues. Therefore, the density and speed of sound of body fluids are close to those of water^18^. We use the average dielectric permittivity of water in the visible and near-IR spectral ranges *ε*_water_ = 1.75 18. The Drude model 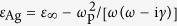 is required to simulate the optical response of silver in the visible and near-IR spectral ranges^19^. We implement the Drude model in the time domain by using the standard auxiliary differential equation method of treatment of the Drude dispersion^19^. The parameters of the Drude model for silver are: *ε*_∞_ = 4.96, *ℏω*_*p*_ = 9.54 eV and *ℏγ* = 0.055 eV^20^.

Simulations of the optical response of the NA and design optimisation require a numerical method capable of tackling geometries with interfaces between materials with disparate dielectric permittivities of silver and water. The 3D optical finite-difference time-domain (Yee-FDTD) method is one of the numerical methods that offers this functionality^19^. The time-dependent Maxwell’s equations (in partial differential form) are discretised using central-difference approximations to the space and time partial derivatives^19^. The resulting finite-difference equations are solved in a leapfrog manner. Firstly, the electric field vector components in a volume of space are solved at a given instant in time. Then the magnetic field vector components in the same spatial volume are solved at the next instant in time. The process is repeated until the desired transient or steady-state electromagnetic field behaviour is fully evolved.

**Figure 1:**
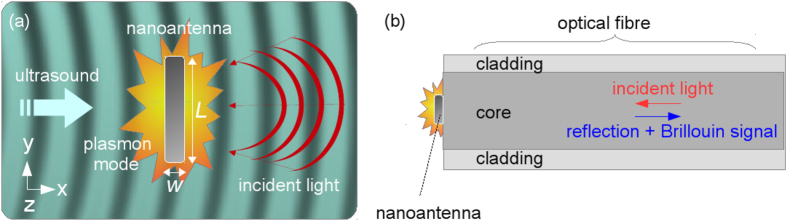
(**a**) Schematic of the reception of ultrasound using a plasmonic NA immersed in water. The big arrow denotes the direction of ultrasound propagation. The acoustic wave in water (blue background) is probed by a higher-order plasmon mode of the NA (denoted by the rectangle). The electric field of this plasmon mode is localised near the surface of the NA. The NA is excited by freely propagating light at the frequency of a higher-order plasmon mode. (**b**) Schematic of a possible embodiment. The NA is fabricated on the edge of an optical fibre (cross-section is shown). The NA is excited by the guided mode of the fibre. The signal reflected from the fibre edge is modulated in response to acoustic pressure variations and is detected by a suitable spectrum analyser (not shown)^14^.

A plane electromagnetic wave propagates along the *x*-axis normally incident on the NA [Fig. 1(a)]. The electric field vector of the plane wave is parallel to the long axis of the NA (the *y*-axis). The spatial resolution step is 2 nm in all coordinate directions, which suffices to accurately model the response of the NA and leads to a ratio of 250 with the shortest optical wavelength considered in the simulations [normally, in photonics accurate results are obtained for the ratio >20 (ref. 19)]. According to the Courant stability condition^19^, the temporal resolution is taken as 3.3 × 10^−12^ *μs*. Optical perfectly matched layers (PMLs) are implemented to avoid unphysical reflections from the boundaries of the optical computational domain^19^. Since PMLs are employed and also because the plasmon modes of the NA are tightly confined to the metal surface, high-accuracy results can readily be obtained with a 2 *μ*m × 2 *μ*m × 2 *μ*m computational domain^19^.

We detect the optical response of the NA in the far-field zone, which is done by implementing a total-field/scattered field (TF/SF) approach and a near-to-far field technique^19^. A field detector is placed in the forward-scattering direction of light by the NA. Electromagnetic field profiles in the near-field-zone of the NA are also recorded. We note that at this stage our model neglects the impact of ultrasound on the optical response of the NA.

**Figure 2:**
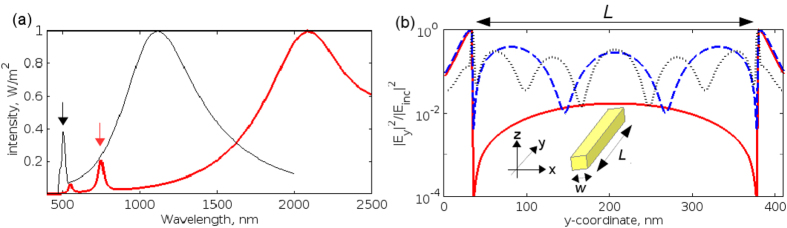
(**a**) Optical response of the NA. Red curve – 3D model. Black curve – 2D model. The vertical arrows indicate the position of the first higher-order plasmon modes in the 2D and 3D spectra. In the 3D model the length of the NA is *L* = 340 nm and its cross-section is *w* = 30 nm. In the 2D model the NA is represented by a plate with 340 × 30 nm^2^ cross-section and infinitely extended in the *z*-direction. (**b**) Electric field intensity traced through the centre of the NA cross-section obtained with the 3D model [red curve in Panel (a)]. The length of the NA *L* = 340 nm is indicated by the double arrow. The red solid curve denotes the profile for the fundamental mode at 2100 nm. The blue dashed (black dash-dotted) curve denotes the profile of the first (second) higher-order mode at 750 nm (550 nm). The 2D simulations produce qualitatively the same profiles for the fundamental and the higher-order modes.

The red curve in Fig. 2(a) shows the far-field optical response of the NA obtained by means of 3D simulations. One can see a broad dipole mode peak centred at ∼2100 nm and two narrow higher-order mode peaks at 750 nm and 550 nm. Although in our further analysis we will be interested in the fundamental and the first higher-order modes only, in Fig. 2(b) we analyse the field profiles corresponding to all modes observed in Fig. 2(a). These profiles show the normalised electric field intensity traced through the centre of the NA cross-section along the *y*-axis. We observe that the fundamental mode has maxima near the edges of the NA, which is a typical picture for a dipolar mode^2^. The profiles of the higher-order modes exhibit additional maxima across the lengths of the NA, which are typical of modes exhibiting a Fabry-Perot-like behaviour^17^. The first higher-order mode has three additional maxima, but the second higher-order mode has five maxima. Because the NA senses the ultrasound by means of its near-field-zone electric fields, in principle the second higher-order mode could also be sensitive to ultrasound. However, the intensity of the second higher-order mode is ∼10 times smaller than that of the first higher-order mode [Fig. 2(a), red curve], which makes it impractical to employ this mode in the hydrophone.

### 2D model of the nanoantenna

Typical 3D simulations of the optical response of the NA do not incur considerable CPU time and memory consumption. However, 3D simulations of the BLS effect are impractically expensive even in terms of supercomputing resources because they combine the optical model of the NA with its acoustic model. Light and ultrasound have very disparate frequency and speed. Hence, the model effectively has two very different time scales. The first time scale is the rapidly varying optical frequency. The second time scale is the slowly varying acoustic frequency. As a result, the optical solution reaches a steady state before the acoustic state has changed substantially due to the propagation of ultrasound.

It is noteworthy that 2D simulations of the NA give a qualitatively similar spectrum to the full 3D approach [Fig. 2(a), black curve]. In this case, the NA is modelled as a silver plate with the 340 × 30 nm^2^ cross-section and infinite length along the *z*-direction. The remaining parameters of our 2D Yee-FDTD model are the same as in the 3D Yee-FDTD model described above. One can see that the 2D model reproduces the line shapes of the dipolar and the first higher-order modes, but the resonance frequencies of the modes are blue-shifted and their relative intensities are different from the prediction of the 3D model. The second higher-order mode is also produced by the 2D model, but it does not appear in Fig. 2(a) because it is blue-shifted to the UV spectral range.

The spectral changes due to the 2D approximation are well-known^21^. However, the discrepancy with the 3D model is unimportant for our analysis because we investigate the difference in the detection of ultrasound by tuning the NA on the dipolar and the first higher-order modes. Indeed, we established that the electric field profiles produced for the dipolar and the higher-order mode by means of 2D simulations are in very good qualitative agreement with the 3D-simulated profiles shown in Fig. 2(b). It is also well-known that plates with acoustically small dimensions exhibit a behaviour similar to vibrations in acoustically small rods^22^,^23^. Thus, without losing important physics of ultrasound scattering, a 3D acoustic problem can also be reduced to a two-dimensional (2D) problem, in which the NA is represented by a plate made of silver and the plane stress approximation is used^24^. We will return to this discussion below.

Significantly, both 2D optical and 2D acoustic simulations are considerably less computationally demanding than the respective 3D simulations. This makes the problem of BLS from ultrasound in the presence of the NA tractable when using available computing resources. Thus, unless otherwise indicated, in what follows we will model the optical and acoustic response of the NA in the 2D approximation of the 3D problem.

## Acoustic Properties of the Nanoantenna

The propagation of ultrasound is a nonlinear process that may lead to the deformation of the incident wave and the generation of new waves at the integer harmonics of the incident frequency^25^. Hence, the equations describing the propagation of ultrasound are intrinsically nonlinear^25^. However, the linearisation of these equations is a useful approximation that is often employed to facilitate understanding the fundamentals of acoustic wave propagation and scattering without losing important physics of light-ultrasound interaction^26^. Therefore, we use the linearised acoustic model to analyse the properties of the NA and demonstrate the operation of the NA hydrophone in the linear acoustic regime. Furthermore, in the Supplementary Information we introduce a model that allows taking into account the nonlinear acoustic effects. We use this model to discuss the impact of the nonlinear effects on the BLS spectra detectable using plasmonic NA hydrophones.

We assume that the major physical effects that contribute to the BLS response are only due to the acoustic pressure that ultrasound exerts on the NA immersed in water. These effects are: (i) deformations of the nanorod and (ii) perturbations of the dielectric permittivities of silver and water. To investigate these effects one has to solve the purely acoustic problem of ultrasound interaction with the NA. In addition, we neglect the thermal expansion effect and other effects that may occur due to the interaction of light with the NA, which is warranted as the intensity of light used in BLS simulations has been chosen to be low enough to avoid such effects^14^.

To conduct acoustic simulations we need a numerical method capable of tackling geometries with interfaces between a fluid (water) and an elastic solid (silver). The elastodynamic finite-difference time-domain (Virieux-FDTD) method is one of the numerical techniques that offers such functionality^27^,^28^,^29^. Conceptually, the Virieux-FDTD method is similar to the Yee-FDTD method, but it is based on the following 2D equations of elastodynamics^27^:


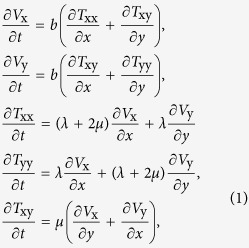


where *b* is the lightness (the inverse of density), *V*_x_ and *V*_y_ are the components of the 2D displacement velocity vector, and *T*_xx_, *T*_yy_, and *T*_xy_ are the components of the stress tensor. The material properties of water are^18^: density *ρ*_water_ = 1000 kg/m^3^ and longitudinal speed of sound *v*_1,water _= 1540 m/s. The material properties of silver are^18^: density *ρ*_Ag_ = 10490 kg/m^3^, longitudinal speed of sound *v*_1,Ag_ = 3650 m/s, transversal speed of sound *v*_t _= 1610 m/s, Young’s modulus *E* = 83 GPa, shear modulus *G* = 30 GPa, and Poisson ratio *v* = 0.37. The Lamé parameters *λ* and *μ* that enter Eq. 1 can be determined from these material constants^24^. Phenomena such as reflection, refraction, and mode conversion at fluid-solid interfaces are fully taken into account by Eq. 1. Absorption phenomena such as viscous and viscoelastic effects are neglected, which is a limitation of the software implementation of the Virieux-FDTD method^29^ used in this work. However, we do not expect absorption phenomena to qualitatively affect our analysis. Indeed, concerns regarding the absorption phenomena may arise in the case of high-frequency (45–50 MHz) IVUS imaging. At lower IVUS frequencies (10–20 MHz), which are the case of this work, such phenomena are usually of little or no clinical significance^30^. Also, acoustic absorption losses in water are taken into account in the discussion in the Supplementary Information (see Supplementary Figs S1 and S2).

Acoustic PMLs are implemented to avoid unphysical reflections from the boundaries of the computational domain^28^,^29^. The size of the computational domain is 2 *μ*m × 2 *μ*m. The source of acoustic waves in water is modelled by a broadband longitudinal velocity pulse centred at 10 MHz and incident in the direction normal to the NA (along the positive *x*-coordinate). In water this source produces a peak acoustic pressure *P* = 5 MPa. Importantly, the spatial resolution step used in the 2D Virieux-FDTD method is the same as in the 2D optical Yee-FDTD simulations: 2 nm, which is done to obtain an accurate model of the NA as well as to enable the translation of data obtained with the Virieux-FDTD algorithm to the Yee-FDTD algorithm in the modelling of the BLS effect (see below). However, the drawback of such a fine spatial resolution is a ratio of 77000 with the shortest acoustic wavelength, which is very large as compared with conventional acoustic simulations (e.g., 8 in ref. 28) and also is the reason for which standard 3D acoustic simulations of the NA would be very time consuming.

However, a large wavelength-to-spatial-step ratio may be used as an advantage because it leads to a completely different convergence behaviour of the FDTD algorithm as compared with the case of the standard ratio^19^. In electromagnetism, the non-standard Yee-FDTD algorithm often has the prefix “high-definition”, and it represents a quite established research area of computational physics^31^,^32^. A different convergence behaviour of the high-definition FDTD has a well-defined physical background. The spatial step chosen in the high-definition FDTD is several orders of magnitude smaller than the wavelength of the incident wave. The object that scatters the incident wave and the computational domain in the high-definition FDTD are also much smaller than the wavelength. Consequently, there are virtually no resonances in the system and scattering is weak. Due to these specific conditions that are often found in, e.g., biomedical and nano-electronics problems^31^,^32^, the incident wave quickly leaves the computational domain and never returns back because it is absorbed by the boundaries, where the PMLs are imposed. As a result, convergence can be achieved within 1–3 oscillation periods of the incident wave, but in some cases it may be enough to run the simulation for less than one period^31^,^32^. Note that this is only valid when the wavelength of the incident wave is much larger than the spatial step. If the wavelength-to-spatial-step ratio is standard (e.g., ref. 20), then resonances and strong scattering become possible and many more oscillation periods are required to reach the end of the transient process in the model^19^.

Thus, in our high-definition Virieux-FDTD model according to the Courant stability condition^19^, the temporal resolution is taken as 3.7 × 10^−7^ *μs*. The simulation duration is chosen to be 0.3 *μs*, which equals three periods of the source field at 10 MHz. As discussed above, the chosen number of periods suffices to obtain converged and accurate results by using the high-definition Yee-FDTD method^31^,^32^. The same number of periods produces a converged and accurate result using the Virieux-FDTD method (see Supplementary Fig. 3), because the Yee-FDTD method and the Virieux-FDTD method are based on the same mathematical paradigm, and thus they have the same convergence and accuracy.

### Vibrations of the nanoantenna

Several observations need to be made before we discuss the results of simulations. We have already noted that the NA is an acoustically small body, for which theory predicts weak scattering–ultrasound bends around the NA without significant changes in the amplitude and phase^33^,^34^,^35^. Thus, the NA becomes subjected to a quasi-uniform compression (in the *x*- and *y*-directions in the case of the 2D model), which warrants the use of the quasi-static approximation^6^,^36^,^37^. In this approximation, the NA is considered as a bulk medium in which *T*_xx_ = *T*_yy_ = −*P* and *T*_xy_ = 0, where *P* is the acoustic pressure in water near the surfaces of the NA^24^. Finally, we used the theory from^38^ to calculate the frequencies of eigen-vibrations of silver nanocubes with the side length 340 nm and 30 nm, and we revealed that the frequencies of the lowest vibration modes equal 2 GHz and 26 GHz, respectively. Thus, the eigen-vibrations of our NA cannot be excited by 10 MHz ultrasound.

**Figure 3:**
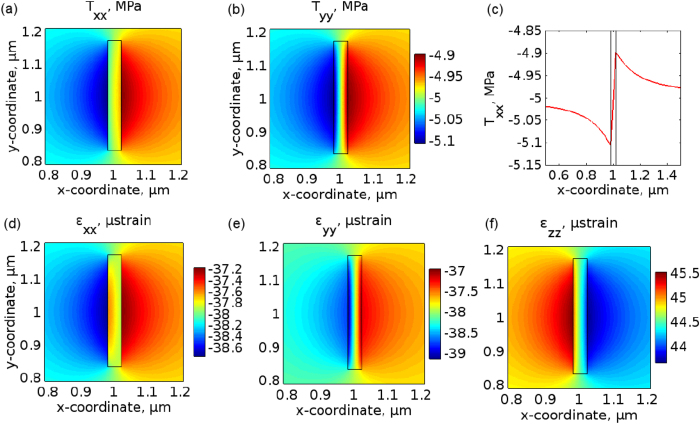
Simulated amplitude of the stress components (**a**) *T*_xx_ and (**b**) *T*_yy_ obtained using the 2D-model. The NA is situated in the centre of the stress profiles, as denoted by the rectangle. Both panels share the same colour bar. The ultrasound propagates along the positive *x*-coordinate direction. The coordinate origin is not (0, 0) because the actual size of the computational domain around the NA is larger than shown. The full computational domain is shown in Supplementary Fig. S2. (**c**) The amplitude of *T*_xx_ across the *x*-coordinate direction for *y* = 0. The region inside the NA is outlined by the two vertical lines. Simulated amplitude of the strain components (**d**) *ε*_xx_, (**e**) *ε*_yy_, and (**f**) *ε*_zz_. Note the different colour bars for the panels (**d**–**f**).

Figure 3(a,b) show the amplitude of the 2D-model simulated stress components *T*_xx_ and *T*_yy_. The shear stress produced in simulations is *T*_xy_ = 0 (to machine accuracy). Outside the NA (in water) the spatial distributions of *T*_xx_ and *T*_yy_ are identical, as it should be in a fluid^24^. As seen along the increasing *x*-coordinate, in front of (behind) the NA the pressure increases (decreases) by ∼2% with respect to the −5 MPa level, which is opposite to the pressure of the incident acoustic wave in water. In general, the amplitude of ultrasound in front of a metal scatterer increases due to local reflection, but behind the scatterer the pressure decreases due to the shadowing effect of the scatterer^26^.

If the dimensions of the NA were comparable with the acoustic wavelength, the pressure in front of the NA would be up two times larger than that of the incident wave^26^. However, in our case the reflection from the NA is low because we deal with an acoustically small object^33^,^34^,^35^. Another possible cause of the low reflection is the excitation of surface Scholte waves, which propagate at interfaces between a fluid and an elastic solid medium and exhibit a behaviour of Rayleigh waves in a fluid-solid half-space^39^,^40^. In ref. 35 it was established that due to the excitation of Scholte waves the reflection of ultrasound from an elastic metal plate immersed in water is very low (≤5%).

A Scholte wave carries almost all of its energy in the fluid rather than in the solid^39^, and its amplitude decreases exponentially in the fluid, but rapidly decays in the solid leading to the formation of an antisymmetric stress profile with respect to the plate plane^39^,^40^. These properties are accurately reproduced by our simulations–the amplitude of *T*_xx_ [Fig. 3(c)] (as well as of *T*_yy_) exponentially decays in water and asymptotically approaches −5 MPa as the distance from the NA increases. The latter confirms the theoretical prediction that ultrasound bends around the NA and far from the NA the amplitude of the scattered wave equals the amplitude of the incident wave^33^,^34^,^35^. Inside the NA the amplitude of *T*_xx_ and *T*_yy_ rapidly decreases to the −5 MPa level predicted of the quasi-static approximation. Because of the continuity of stress outside and inside the NA, this behaviour leads to the formation of an antisymmetric stress profile [Fig. 3(c)], which is also predicted by theory^40^.

Next, the Hooke’s law in the plane stress approximation^24^ is used to predict the deformations of the NA caused by the stress:


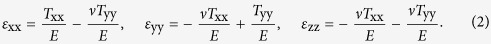


Figure 3(d–f) show the amplitude of the strain components *ε*_xx_, *ε*_yy_, and *ε*_zz_ obtained using the 2D model. A negative (positive) normal strain implies that an object is compressed (stretched) due to acoustic pressure. Thus, we observe negative values of *ε*_xx_ and *ε*_yy_ because the acoustic pressure compresses the NA along the *x*- and *y*-coordinate directions. In the plane stress approximation, Hooke’s law also predicts an elongation of the NA in the *z*-direction, which is reproduced by our simulation as the positive values of the *ε*_zz_ component. This component is not zero because of Poisson’s effect^24^: the plate is free to expand in the *z*-direction as it is compressed in the *x*- and *y*-directions, and there is no out-of-plane constraint to prevent this.

When ultrasound propagates in a metal, the crystalline lattice is periodically deformed^41^,^42^. These deformations may lead to changes in the dimensions of the NA and density of the constituent metal. The latter also results in perturbations of the dielectric permittivity of the metal. However, noticeable deformations occur only when the stress amplitude is large: ∼100 MPa for aluminium and copper^41^,^42^. Here we refer to reported data for aluminium and copper^41^,^42^ because data for silver are unavailable, but the elastic properties and the lattice parameter of bulk aluminium and copper (*E* = 69 GPa and *a* = 4.05 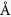 for aluminium, and *E* = 120 GPa and *a* = 3.62 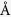 for copper) are similar to those of bulk silver (*E* = 83 GPa and *a* = 4.09 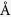)^18^.

The strain induced by ultrasound in the NA along the *x*- and *y*-directions equals ∼−38 *μ* strain. This implies that the total length of the NA is decreased by Δ*L* ≈ 0.13 Å, which is calculated as the strain times the NA length. Accordingly, the width of the NA is decreased by Δ*w* ≈ 0.01 Å. Considering the lattice parameter *a* of the constituent metal, for the same value of the strain we obtain the relative change Δ*a*/*a* ≈ −0.004% whose role will be clarified later on.

In our simulations of the BLS effect, it is impossible to take such a small deformation of the NA into account by either using a very fine mesh or applying a technique from ref. 43. the extension of which to the case of plasmonic nanostructures has proven to be challenging. However, such simulations may be circumvented because it can be shown that the observed deformation of the NA by ultrasound is negligibly small and thus cannot significantly contribute to the BLS response. In refs 41 and 42 it is shown that in aluminium and copper the relative change in the lattice parameter is zero if the pressure amplitude equals 2.4 MPa. A small value Δ*a*/*a* ≈ −0.004% produced by our simulations for silver is in reasonable agreement with the data for aluminium and copper. This implies that in our case the pressure of ultrasound is not high enough to induce structural changes in silver. Indeed, noticeable deformations Δ*a*/*a* ≈ 0.02–0.03% in aluminium and copper occur only at significantly higher amplitudes of ∼100 MPa^41^,^42^, which are also expected to produce comparable deformations of the lattice parameter of silver. However, such amplitudes are not used in IVUS/IVPA imaging and thus they will not be considered in our work. (We note though that large amplitudes are employed in high-intensity focused ultrasound (HIFU), which is an evolving medical technology for noninvasive surgery and cancer therapy^44^). Therefore, in our further analysis it is safe to assume that the changes in the shape of our NA are negligibly small.

### Perturbations of the dielectric permittivity

Perturbations of the dielectric permittivity of the medium originate from alternating density changes caused by acoustic pressure that compresses and decompresses the medium^3^. We calculate the perturbations of the dielectric permittivity by using the values of the displacement field **u**(*x*, *y*, *t*) within the media (water and silver)^43^:


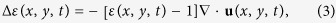


where *ε*(*x*, *y*, *t*) is the unperturbed dielectric permittivity of the medium and the divergence of the displacement field 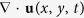 equals the trace of the strain tensor^24^.

In analytical analysis of ultrasound propagation in water it is common to assume that water is incompressible (the Poisson’s ratio *v* = 0.5)^18^. However, this is not the case of our numerical model because water, as well as biological tissues and bodily fluids with high water content^45^, are in fact only nearly incompressible materials^46^. Indeed, water has a finite bulk modulus 2.15 GPa, and the inverse of the bulk modulus gives the water’s compressibility^18^. For example, at the hydrostatic pressure 40 MPa at a depth of ∼4000 m the volume compression of ocean water is ∼1.8%^46^. Consequently, to calculate the dielectric permittivity perturbations of water, in Eq. 3 we employ a well-established approach to take the empirical Poisson’s ratio of water *v* = 0.495^45^. This choice also allows avoiding well-known numerical instabilities often arising in modelling of incompressible media^47^.

In our model, it is advantageous to employ Eq. 3 and not to rely on the use of tabulated photoelastic (elasto-optic) coefficients^48^ (as was done in the previous theory^6^). This is because the literature sources on electrostriction and photoeleasticity (the electrostrictive tensor is closely linked to the photoelastic tensor^49^) give scarce information about the electrostrictive and the photoelastic coefficients in metals^50^,^51^. Indeed, the electric field strength inside a metal is zero and it has a little effect on the properties of the metal. Consequently, the interest in electrostriction in metals is mostly of theoretical nature, but experimental data are sparse and difficult to obtain^51^.

Moreover, in order for our discussion to be complete, it is essential to know the photoelastic coefficients of silver and water in both the visible and near-infrared spectral ranges. However, even for the most common optical dielectric materials tabulated experimental data are mostly only available in the visible spectral range^48^. This is a problem for the previous theory relying on the use of photoelastic coefficients, and this drawback was pointed out in a relevant discussion^6^.

In simulations of the optical response of the NA (Fig. 2), the dielectric permittivity of water is constant at all frequencies. Here, we use this value in Eq. 3 to calculate Δ*ε*(*x*, *y*, *t*). However, the frequency-dependent dielectric permittivity of silver is modelled by using the Drude model 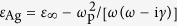. In Eq. 3 we use *ε*_∞_ of the Drude model as the unperturbed dielectric permittivity of silver. This is done by analogy with the previous models^20^,^52^ in which the value of *ε*_∞_ fluctuates when the dielectric function of metal is changed due to nonlinear optical effects, but the other term of the Drude formula 
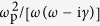
 remains unchanged. The latter assumption is correct because negligibly small changes in the shape of our NA are not expected to affect the value of *ω*_p_^53^.

**Figure 4:**
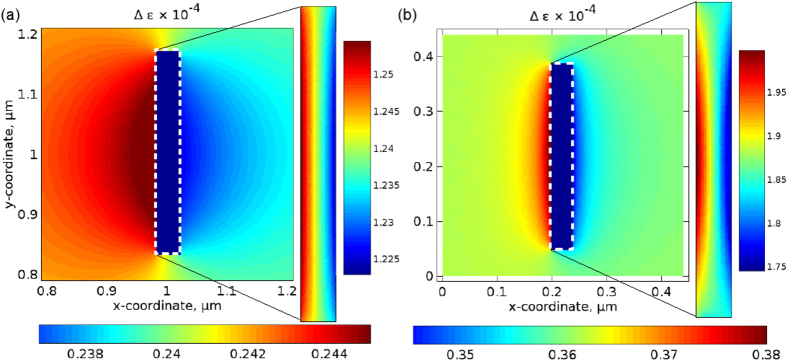
Amplitude of the perturbations of the dielectric permittivity Δ*ε* due to acoustic pressure 5 MPa calculated by using (**a**) the 2D model and (**b**) 3D model (the profile is shown in the *x*-*y* cross-section of the NA). Main panels: perturbation profile in water. The NA is situated in the centre. Inset: close-up of the perturbation profile inside the NA. Note the different colour bars for the profiles in water and silver. As can be seen by comparing the respective vertical and horizontal colour-bar scales, in the 2D model [panel (a)] the amplitude of Δ*ε* is ∼1.5 times smaller than in the 3D model [panel (b)]. See Supplementary Fig. S4 online for more details. In panel (a), the coordinate origin is not (0, 0) because the actual size of the computational domain around the NA is larger than shown.

Figure 4(a) shows the profiles of Δ*ε*(*x*, *y*, *t*) in water and in the NA calculated by using the 2D model. The amplitude of the dielectric permittivity perturbations in silver is Δ*ε* ≈ 1.25 × 10^−4^. In water we obtain Δ*ε* ≈ 0.245 × 10^−4^. In agreement with Eq. 3, the dielectric permittivity perturbation profiles follow the spatial distribution of the strain. Thus, in water the perturbations extrema are observed in close proximity to the surfaces of the NA. Inside the NA, the perturbations extrema are located near the surfaces and the amplitude of Δ*ε* linearly decreases in the direction away from the surfaces. The perturbations in water and silver contribute to the BLS response of the NA and they are taken into account in the following section.

## Plasmon-enhanced Brillouin Light Scattering

In this Section, we simulate the effect of BLS from ultrasound in the presence of the NA. We demonstrate that the NA tuned to its first higher-order plasmon mode is more sensitive to ultrasound as compared with the same NA tuned on the fundamental dipolar mode.

As a reference, we also calculate the BLS scattering from ultrasound propagating in bulk water (without the NA). In this case, the dielectric permittivity perturbations of water are viewed by the incident light wave as a moving diffraction grating. In conventional optical hydrophones, light-ultrasound interaction occurs over the entire length of the underwater laser beam propagation. This length is usually much larger than the wavelength of light, which makes the BLS scattering effect additive and more pronounced^4^. However, in our model light travels through bulk water for a small distance 2 *μ*m, over which the additive effect is negligibly small. This scenario is chosen deliberately because the potential applications of our hydrophone include the operation in small volumes of fluid.

By using the 2D Yee-FDTD method we calculate the spectrum of light that travels through bulk water. The dielectric permittivity fluctuations *δε*(*x*, *y*, *t*) are taken into account as a perturbed dielectric permittivity 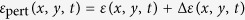, where *ε*(*x*, *y*, *t*) is the unperturbed dielectric permittivity and the value of Δ*ε*(*x*, *y*, *t*) is obtained in 2D Virieux-FDTD simulations and transferred to 2D Yee-FDTD method. This transfer of data is not challenging thanks to our choice of the same spatial discretisation in both algorithms. On average, a single simulation run takes about 48 hours of 16-core CPU time.

**Figure 5:**
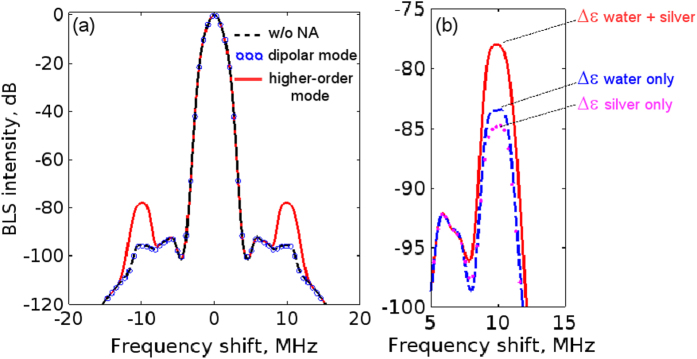
(**a**) Simulated BLS intensity spectra as a function of the frequency shift. The 2D model was used. Black dashed curve: the BLS spectrum in bulk water (without NA) at the resonance wavelength of the first higher-order mode. This spectrum is virtually the same at the resonance wavelength of the dipolar mode. Red solid curve: the BLS spectrum in the presence of the NA at the frequency of the higher-order mode. Note that from −5 MHz to 5 MHz the black dashed curve and red solid curve coincide to graphical accuracy. Open circles: the BLS spectrum in the presence of the NA at the frequency of the dipolar mode. Note the dips around the frequency shift ±4.5 MHz that arise from the Blackman-Harris apodisation function and are irrelevant to the discussion. (**b**) Close-up of the Brillouin peak in the presence of the NA (the results for bulk water are not shown). Solid red curve: combined effect of the perturbations Δ*ε* in water and silver. Dashed blue curve: only perturbations of the dielectric permittivity of water are considered in the model. Dotted magenta curve: only perturbations of the dielectric permittivity of silver are considered in the model.

For the case of bulk water (without NA) we conduct two simulations in which the wavelength of the incident light corresponds, respectively, to the resonance wavelength of the dipolar mode (1120 nm) and the first higher-order mode (505 nm) in the 2D model of the NA [Fig. 2(a)]. The Blackman-Harris apodisation function^54^ is used to improve the resolution of the resulting spectra. We observe that in bulk water the spectra for the two wavelengths coincide to graphical accuracy [Fig. 5(a), black curve]. Moreover, at both wavelengths of the incident light the Brillouin peaks are very weak because the dielectric permittivity perturbations of water caused by 5 MPa ultrasound are very small and, most significantly, there is no additive effect of light-ultrasound interaction at the propagation length 2 *μ*m.

In the presence of the NA [Fig. 5(a), red curve] the simulation reveals two Brillouin peaks (Stokes and anti-Stokes components) situated on the shoulders of the central Rayleigh peak and shifted from the Rayleigh peak by the frequency of ultrasound (±10 MHz). As compared with the intensity of the BLS signal in bulk water, at the frequency shift ±10 MHz we observe a ∼60-fold increase in the intensity of the Brillouin peaks with respect to their intensity in the case of bulk water. The impact of the NA on the BLS spectrum is negligible at the other frequencies.

The increase in the BLS intensity at ±10 MHz is attributed to the Fabry-Perot-like behaviour of the NA when it is tuned on its first higher-order plasmon resonance. This is confirmed by means of simulations in which the NA is tuned to its dipolar mode. As shown in Fig. 5(a) by the open circles, there is no visible BLS intensity changes as compared with the case of BLS in bulk water.

Our simulations also allow us to investigate the relative impact of the perturbations of the dielectric permittivity of water and silver. We conduct two additional simulations in which we neglect the perturbations in silver or water, respectively. In Fig. 5(b) we observe that the intensity of the Brillouin peaks is ∼1.5 times more sensitive to the perturbations in water than in silver. Although the perturbation amplitude in silver is five times larger than in water, this result is consistent with the fact that the electric field of the higher-order plasmon mode of the NA is mostly localised at the water-silver interface and its penetration into metal is small [Fig. 2(b)].

## Further Validation of the 2D model by 3D Acoustic Simulations

Finally, we demonstrate how the overall result has been affected by the reduction of the NA model from 3D to 2D. Here, estimations are required because the full 3D problem of light scattering from ultrasound in the presence of the NA cannot be solved numerically due to a severe CPU time penalty. The incapability to solve a similar 3D model was realised in the previous works and a simplified model based on the quasi-static approximation of ultrasound effect was proposed to investigate acoustically small micro-ring optical resonators acting as ultrasound sensors^6^. It follows from the cited paper that the proposed model produces satisfactory agreement with experimental results. However, the validity of the quasi-static approximation remains unjustified by means of rigorous numerical simulations.

The gap in confirming the validity of the quasi-static approximation is partially bridged in our work. Although full 3D simulations of light scattering from ultrasound in the presence of the NA are still impossible in our model, we conduct 3D acoustic simulations by means of a 3D Virieux-FDTD method^55^ that relies on the same model equation as the 2D method described above^29^. We note that in our 3D simulation the size of computational domain is 0.44 *μ*m × 0.44 *μ*m, which is smaller than in the 2D model (2 *μ*m × 2 *μ*m) and it is done to reduce computational efforts arising due to the addition of one more spatial coordinate. However, we keep the same material and model parameters used in the 2D model, including the spatial and time steps, and the total simulated time. The convergence of the 3D Virieux-FDTD method is discussed in the Supplementary Information (see Supplementary Fig. S3) and it is the same as in the 2D case. Thus, we create conditions for a quantitative comparison of the results obtained using the two models.

We start with the 3D quasi-static approximation model, in which the stress −*P* is created by ultrasound at all surfaces of the NA. By using the generalised Hooke’s law^24^ we obtain the strain ∼−15 *μ* strain in all coordinate directions. This value is about two times lower than that predicted by the 2D model, which implies that our conclusion about the unchanged shape of the NA should also hold in the 3D case because the relative change in the lattice constant is Δ*a*/*a* ≈ −0.0015%. As for the change in the dielectric permittivity, for silver NA we predict the amplitude Δ*ε* ≈ 1.86 × 10^−4^.

**Figure 6:**
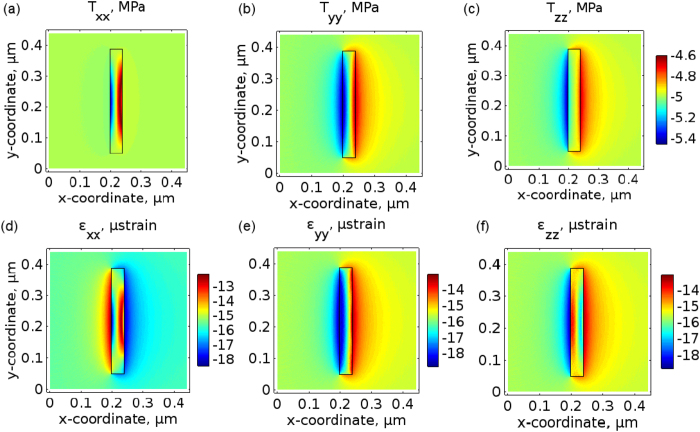
Simulated amplitude of the stress components (**a**) *T*_xx_, (**b**) *T*_yy_, and (**c**) *T*_zz_ plotted in the *x*-*y* cross-section of the NA. The 3D model was used. The NA is situated in the centre of the stress profiles, as denoted by the rectangle. Panels (a–c) share the same colour bar. The ultrasound propagates along the positive *x*-coordinate direction. Simulated amplitude of the strain components (**d**) *ε*_xx_, (**e**) *ε*_yy_, and (**f**) *ε*_zz_. Note the different colour bars for the panels (d–f).

The predictions of the simple quasi-static model are in excellent agreement with the result obtained using the 3D acoustic model (Fig. 6). From Fig. 6(a–c) one can also see that the 3D-simulated profiles of the stress components (plotted in the *x*-*y* cross-section of the NA) are in good agreement with the respective 2D-simulated profiles in Fig. 3(a,b). This good agreement between the 3D and 2D-simulated profiles confirms the previous analytical theories^22^,^23^ suggesting that acoustically small rods behave similar to acoustically small plates. Significantly, it also justifies the simplification from the 3D model to the 2D model. We note that the magnitude of the 3D-simulated strain components [Fig. 6(d–f)] is close to −15 *μ* strain that is the value predicted by the quasi-static model. What is not predicted by the quasi-static model is the excitation of a surface Scholte wave at the water-silver interfaces, which leads to variations in the strain magnitude near the edges of the NA. In the 3D model these variations are slightly stronger than in the 2D model. This is consistent with the fact that in the 3D model the acoustic waves interacts with the additional sharp interfaces of the NA as compared with the 2D model, and these additional interfaces give rise to stronger surface waves^56^.

Using the same procedure as above, we calculate the perturbations of the dielectric permittivity in silver and water, but now we rely on the data obtained using the 3D acoustic model. The resulting Δ*ε* profile plotted in the *x*-*y* cross-section of the NA [Fig. 4(b)] is qualitatively very similar to the profile obtained using the 2D data [Fig. 4(a)], which is especially the case of silver [compare the insets in Fig. 4(a,b)]. We reveal, in excellent agreement with the prediction of the quasi-static model, that in silver Δ*ε* ≈ 1.86 × 10^−4^ in the middle of the NA. In water, far from the edges of the NA Δ*ε* ≈ 0.36 × 10^−4^. Both in silver and water, near the edges of the NA the value of Δ*ε* slightly deviates from the prediction of the quasi-static model. This is because, similar to the behaviour observed in the 2D case [Fig 4(a)], the values of the stress and strain are affected by the excitation of a surface Scholte wave at the water-silver interface.

Thus, the profile of the dielectric permittivity perturbations Δ*ε* produced by using the simplified 2D acoustic model is qualitatively similar to the profile obtained using the 3D acoustic model. However, in the 2D model the amplitude of Δ*ε* of both silver and water is ∼1.5 times smaller than in the 3D model (compare the colour-bar scales in Fig. 4(a,b), and also see Supplementary Fig. S4). This difference is consistent with the fact that in the 2D model the surfaces of the NA perpendicular to the *z*-axis are effectively stress-free, but in the 3D case the stress is applied to all surfaces. Consequently, the overall result predicted by the 2D model of light interaction with ultrasound in the presence of the NA (Fig. 5) also underestimates the sensitivity of the NA to ultrasound. Indeed, it is well-known (ref. 3) that the total intensity of light *I* scattered from ultrasonic waves propagating in fluids is related to the departure of the dielectric permittivity of the medium in a small volume from its average value as 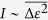.

## Conclusions

We have theoretically demonstrated the possibility to employ an ultrasmall plasmonic NA as an optical ultrasonic hydrophone. We have shown that the exploitation of a higher-order plasmon mode of the NA leads to increased sensitivity to ultrasound. This effect originates from the near-field enhancement of electric field due to the higher-order plasmon resonance of the NA, and it is not observed when the fundamental dipolar mode is excited. The proposed NA-based ultrasound reception mechanism can be used in various modalities of biomedical ultrasound imaging, including intravascular imaging of small blood vessels. Also, the NA can help to additionally increase the previously demonstrated benefits of the BLS spectroscopy technique from plasmon resonances in metallic nanostructures.

## Additional Information

**How to cite this article**: Maksymov, I. S. and Greentree, A. D. Plasmonic nanoantenna hydrophones. *Sci. Rep.* **6**, 32892; doi: 10.1038/srep32892 (2016).

## Supplementary Material

Supplementary Information
