# Supplementary material for: Plasmonic nanoantenna hydrophones
Source: Sci Rep. 2016 Sep 9;6:32892. doi: 10.1038/srep32892 (PMC5017197; doi:10.1038/srep32892)
Supplement: Supplementary Information [file srep32892-s1.pdf]

Supplementary Information for: 'Plasmonic nanoantenna hydrophones'

Ivan S. Maksymov and Andrew D. Greentree
ARC Centre of Excellence for Nanoscale BioPhotonics,
School of Science, RMIT University, Melbourne, VIC 3001, Australia

Introduction

In the main text, our model is based on the linearised equations describing the propagation of ultrasound in water and silver. This model also neglects the absorption of ultrasound by the medium.

However, in practice, the propagation of ultrasound is a nonlinear process that may lead to the deformation of the incident wave and the generation of new waves at the integer harmonics of the incident frequency [S1]. These harmonics may be detected by means of the BLS from ultrasound [S2]. In this case, the spectrum of the scattered light will consists of *multiple* Brillouin peaks that correspond to all acoustic waves propagating in the medium. These peaks will be shifted from the optical frequency by the frequencies of the first and higher-order harmonic waves [S3]. Significantly, the optical intensity of the BLS peaks will depend on the amplitude of the corresponding acoustic waves [S2, S3].

In the scenario of the NA immersed into water, the impact of the acoustic nonlinear effects will depend on the distance travelled by ultrasound in water [S1] and the acoustic scattering properties of the NA [S4]. Based on the results obtained in the main text, it is safe to exclude the impact of the NA, because the NA behaves as an acoustically small body and its acoustic scattering cross-section is negligibly small. Furthermore, it has been demonstrated that changes in nonlinear acoustic interaction become apparent only in strongly-scattering media such as suspensions of rigid nanoparticles [S4].

We also assume that the distance travelled by ultrasound does not exceed 1 mm, which is a reasonable approximation for IVUS/IVPA imaging of small diameter blood vessels (< 0.5 mm), which is one of the applications of the plasmonic NA hydrophone envisioned in the main text. It is noteworthy that this propagation distance is smaller than the discontinuity formation distance, which is an important parameter of nonlinear acoustics. The discontinuity formation distance may also be defined as the propagation length that gives rise to the second harmonic amplitude that formally attains 1/2 of the first harmonic amplitude [S5]. By keeping the propagation length below the discontinuity formation length, in our model we avoid the appearance of strong acoustic nonlinearities such as the formation of shock waves.

Thus, we solve a problem of nonlinear acoustic interaction in water in order to predict the impact of the acoustic nonlinear effects on the BLS response of the plasmonic NA hydrophone. As a result of the approximations justified above, this problem becomes tractable in the one-dimensional (1D) space by solving the model equations of nonlinear acoustics [S1]. Although analytical solutions to these equations are known in the lossless approximation [S5], in the presence of losses they are either difficult or non-existing. Therefore, we employ the numerical FDTD method [S6].

Model equations of nonlinear acoustics

The propagation of ultrasound in water is governed by the equations of nonlinear acoustics [S1, S7]. We begin with the continuity equation, which links the vector velocity vector $\boldsymbol{v}$ to the instantaneous density of liquid ρ as

$$\frac{\partial \rho}{\partial t} + (\boldsymbol{v}\cdot\boldsymbol{\nabla})\rho + \rho\boldsymbol{\nabla}\cdot\boldsymbol{v} = 0, \tag{S1}$$

where t is the time.

The second equation is the momentum equation:

$$\rho\left(\frac{\partial \boldsymbol{v}}{\partial t} + (\boldsymbol{v}\cdot\boldsymbol{\nabla})\boldsymbol{v}\right) = -\boldsymbol{\nabla}P + \eta\nabla^2\boldsymbol{v} + (\zeta + \frac{\eta}{3})\boldsymbol{\nabla}(\boldsymbol{\nabla}\cdot\boldsymbol{v}), \tag{S2}$$

where P is the thermodynamic pressure, η the shear viscosity, and ζ the bulk viscosity.

The third, the thermodynamic equation of state, relates three physical quantities describing the thermodynamic behaviour of the liquid. These quantities can be P, ρ, and the temperature T. Alternatively, they can be P, ρ, and the specific entropy s. Normally, one applies a Taylor expansion about the equilibrium state, which to the second order is

$$p = c_0^2\rho' + \frac{c_0^2}{\rho_0}\frac{B}{2A}\rho'^2 + \left(\frac{\partial P}{\partial s}\right)_{\rho,0} s', \tag{S3}$$

where p, ρ', and s' are the dynamic pressure, density, and entropy, respectively. These quantities describe small disturbances relative to the uniform state of rest. The parameters B/A and c_0 are the nonlinear parameter of the medium and the 'small signal' speed of ultrasound, respectively.

The fourth equation is the entropy equation

$$\rho T\left(\frac{\partial s}{\partial t}+(\boldsymbol{v}\cdot\boldsymbol{\nabla})s\right)=\kappa\nabla^2 T+\zeta(\boldsymbol{\nabla}\cdot\boldsymbol{v})^2+\frac{\eta}{2}\left(\frac{\partial v_{\rm i}}{\partial x_{\rm j}}+\frac{\partial v_{\rm j}}{\partial x_{\rm i}}-\frac{2}{3}\delta_{\rm i,j}\frac{\partial v_{\rm k}}{\partial x_{\rm k}}\right)^2, \tag{S4}$$

where κ is the thermal conductivity and $\delta_{\rm i,j}$ is the delta function. The last term of the right hand side of Eq. S4 is written in Cartesian tensor notation.

By keeping the first and second order terms in Eqs. S1-S4, we obtain the following nonlinear equations. The continuity equation is

$$\frac{\partial\rho'}{\partial t}+\rho_0\boldsymbol{\nabla}\cdot\boldsymbol{v}=\frac{1}{\rho_0 c_0^4}\frac{\partial p^2}{\partial t}+\frac{1}{c_0^2}\frac{\partial\mathcal{L}}{\partial t}, \tag{S5}$$

and the momentum equation is re-expressed as

$$\rho_0\frac{\partial\boldsymbol{v}}{\partial t}+\boldsymbol{\nabla}p=-\frac{1}{\rho_0 c_0^2}(\zeta+\frac{4\eta}{3})\boldsymbol{\nabla}\frac{\partial p}{\partial t}-\boldsymbol{\nabla}\mathcal{L}, \tag{S6}$$

where $\mathcal{L}=\frac{1}{2}\rho_0 v^2-\frac{p^2}{\rho_0 c_0^2}$ is the second-order Lagrangian density. The entropy equation can be simplified to

$$\rho_0 T_0\frac{\partial s'}{\partial t}=\kappa\nabla^2 T, \tag{S7}$$

where T_0 the temperature of the medium at rest. The substitution of Eq. S7 to Eq. S3 and the use of thermodynamic relationships, one can write the equation of state as

$$\rho'=\frac{p}{c_0^2}-\frac{1}{\rho_0 c_0^4}\frac{B}{2A}p^2-\frac{\kappa}{\rho_0 c_0^4}\left(\frac{1}{c_{\rm v}}-\frac{1}{c_{\rm p}}\right)\frac{\partial p}{\partial t}, \tag{S8}$$

where $c_{\rm v}$ and $c_{\rm p}$ are the specific heat capacities at constant volume and pressure, respectively.

Following Ref. [S6], by using the first-order differencing in time and space, we derive the following finite-difference equations for the nonlinear 1D case:

$$v^{\rm n+\frac{1}{2}}(k)=v^{\rm n-\frac{1}{2}}(k)+g_{\rm b}\left[p(k)^{\rm n}-p^{\rm n}(k+1)\right]-\frac{\zeta+\frac{4\eta}{3}}{\rho_0^2 c_0^2\Delta_{\rm z}}\left[p^{\rm n}(k+1)-p^{\rm n}(k)-p^{\rm n-2}(k+1)+p^{\rm n-2}(k)\right], \tag{S9}$$

where the constants c_0, ρ_0, ζ, and η are co-ordinate dependent, and $g_{\rm b}=\Delta_{\rm t}\Delta_{\rm z}/\rho_0$.

$$\begin{aligned} p^{\rm n+1}(k)=-\frac{1}{\delta}\Big\{&\left[c_0^4\Delta_{\rm z}p^{\rm n-1}(k)-2c_0^4\Delta_{\rm z}p^{\rm n}(k)\right]\rho_0 C+c_0^4\Delta_{\rm t}^2\rho_0^2\left[v^{\rm n+\frac{1}{2}}(k-1)-v^{\rm n+\frac{1}{2}}(k)\right]\\ &+c_0^2\Delta_{\rm t}\Delta_{\rm z}p^{\rm n}(k)\rho_0-\beta\Delta_{\rm t}\Delta_{\rm z}p^{\rm n}(k)p^{\rm n-1}(k)+\beta\Delta_{\rm t}\Delta_{\rm z}p^{\rm n}(k)^2\Big\}\end{aligned}, \tag{S10}$$

where $\delta=\left[c_0^4\Delta_{\rm z}\rho_0 C-c_0^2\Delta_{\rm t}\Delta_{\rm z}\rho_0-\beta\Delta_{\rm t}\Delta_{\rm z}p^{\rm n-1}(k)+\beta\Delta_{\rm t}\Delta_{\rm z}p^{\rm n}(k)\right]$, $\beta=1+\frac{B}{2A}$ and $C=\frac{\kappa}{\rho_0 c_0^4}\left(\frac{1}{c_{\rm v}}-\frac{1}{c_{\rm p}}\right)$. Importantly, as compared with the linear FDTD algorithm [S6], the values of pressure p at the two time steps – $p^{\rm n-2}$ and $p^{\rm n-1}$ – must be additionally retained in the computer memory.

Nonlinear acoustic interaction

The following material constants of water at 20° are used in our nonlinear acoustic simulations: the shear viscosity $\eta=1.002\times10^{-3}$ Pa s, bulk viscosity $\zeta=3.09\times10^{-3}$ Pa s, specific heat capacity at constant pressure $c_{\rm p}=4.182\times10^3$ J/(kg K) and constant volume $c_{\rm v}=c_{\rm p}/1.33$, and thermal conductivity $\kappa=0.597$ W/(m K) [S8]. The acoustic nonlinearity of water is taken into account by using a modified nonlinear coefficient $\beta=1+\frac{B}{2A}=3.5$ [S1]. Setting $\beta=0$ converts the nonlinear model into the linear one. The incident monochomatic sinusoidal pressure plane wave has the frequency 10 MHz and amplitude $P=5$ MPa. The time step Δt is calculated using the Courant stability

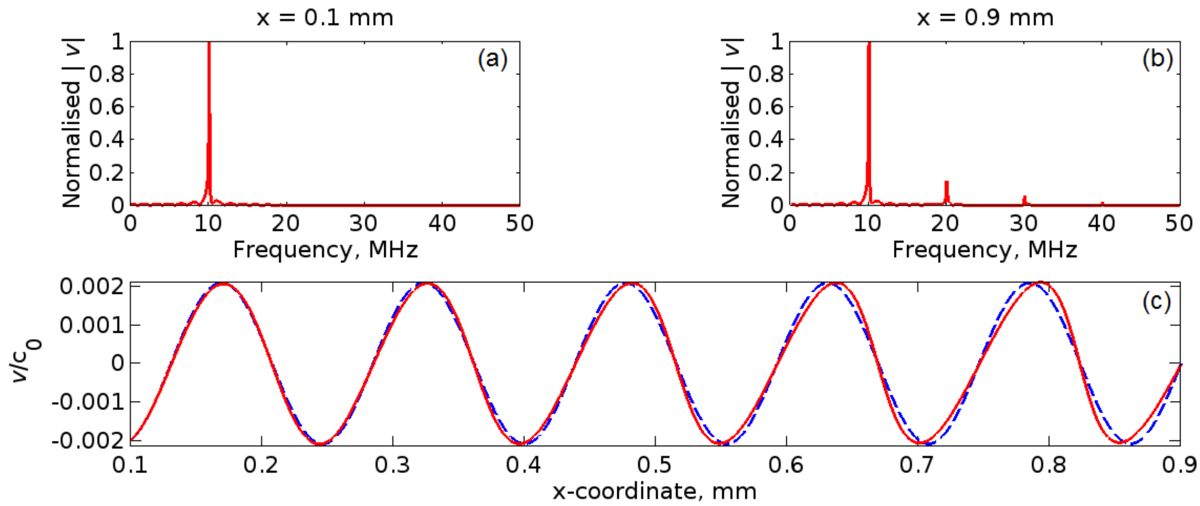

FIG. S1: (a, b) Normalised frequency spectra of the velocity wave for the two propagation distances measured along the x-coordinate: 0.1 mm and 0.9 mm. Whereas the impact of the nonlinear effects on the frequency spectrum is negligible at $x = 0.1$ mm, it leads to the appearance of the higher-order harmonics of the incident wave at $x = 0.9$ mm. (c) Spatial profiles of the velocity wave in the presence of the nonlinear effects (solid line) and without them (dashed line; this line is effectively the profile of the incident wave). The velocity wave amplitude v is normalised on the speed of ultrasound c_0 in water. The appearance of the harmonics in the spectrum manifests itself as the deformation of the wave profile (solid line) as compared with the sinusoidal profile of the incident wave (dashed line).

condition and the spatial discretisation step is $\Delta x = 1$ μm. We also use the first-order Mur's absorbing boundary conditions (ABCs) to avoid unphysical reflections from the boundaries [S6].

Figures S1(a, b) show the frequency spectra of the velocity wave for the two propagation distances measured along the x-coordinate direction: 0.1 mm and 0.9 mm. These spectra were obtained from the simulated time dependencies by means of Fast Fourier transformation.

The impact of the nonlinear effects on the frequency spectrum is negligible at $x = 0.1$ mm. However, at $x = 0.9$ mm we observe not only the the initial frequency 10 MHz, but also its second, third and so on harmonics that have been generated as a result of the nonlinear acoustic interaction. Figure S1(c) compares the spatial profiles of the velocity wave in the presence of the nonlinear effects (solid line) and without them (dashed line). One can see that the harmonic content of the nonlinear wave manifests itself as a slightly deformed wave profile as compared with the sinusoidal profile of the incident wave.

We note that the amplitude of the second (third) harmonic constitutes 15% (6%) of the amplitude of the first harmonic. The amplitudes of the forth and fifth harmonics are even smaller. We remind that in the main text it was established that the the NA is an acoustically small body and it virtually does not affect the propagation of the first harmonic wave. At the frequencies of the second and third harmonics the acoustic wavelength (~ 75 μm and ~ 50 μm, respectively) is two orders of magnitude larger than the dimensions of the NA. Therefore, at these frequencies the NA also behaves as an acoustically small body and all results obtained for the first harmonic remain applicable to the second and third harmonics.

The assumptions above are justified by means of 2D Virieux-FDTD simulations of the amplitude of the stress component T_{xx} at the frequency of the second harmonic 20 MHz. For consistency with the results in Fig. 3(a) in the main text, we maintain the same simulation parameters including the number of oscillation periods and the pressure of the incident wave. As predicted above, at 20 MHz we obtain visually the same spatial profile as in Fig. S2(a), which presents the same result as in Fig. 3(a) in the main text but shows the full computational domain. However, there exists a certain qualitative difference that makes physical sense. This can be seen, for example, by plotting the difference $T_{\mathrm{xx}}^{10\ \mathrm{MHz}} - T_{\mathrm{xx}}^{20\ \mathrm{MHz}}$ between the stress components at the frequencies of the first and the second harmonics [Fig. S2(c)]. To eliminate possible numerical artefact and facilitate comparison between the features of the stress profiles, both stress components have been normalised to the respective stress profiles in water without the NA, calculated at 10 MHz and 20 MHz.

The maximum difference of ± 0.0015 is observed in close proximity to the NA, but this value quickly drops and approaches zero at 1 μm off the NA. This behaviour agrees with the fact that the NA is a poor acoustic scatterer and that only the acoustic fields in close proximity to the NA are disturbed. Moreover, at 20 MHz the scattering of ultrasound by the NA gets slightly stronger as at 10 MHz, because for the 20 MHz wave the NA is a larger scatterer.

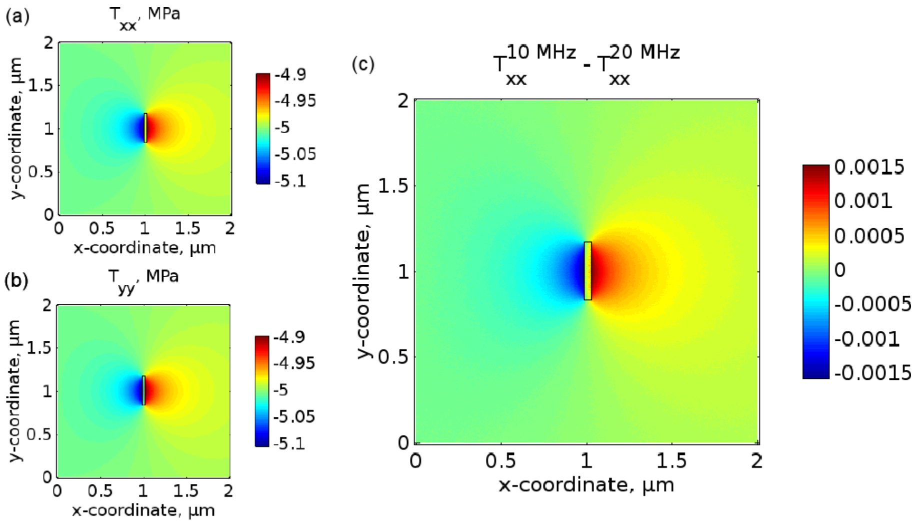

FIG. S2: Amplitude of the stress components (a) $T_{\rm xx}$ and (b) $T_{\rm yy}$ at the 10 MHz frequency of incident acoustic wave. The same result as in Fig. 3(a, b) in the main text is presented, but the full computational domain is shown. The contours of the NA are denoted by the small rectangle in the centre of the profiles. (c) Difference between the normalised amplitudes of the $T_{\rm xx}$ stress components at the 10 MHz frequency of the first harmonic and the 20 MHz frequency of the second harmonic. The ultrasound propagates along the positive x-coordinate direction. All results were obtained using the 2D model.

This leads to an increase in backscattering from the NA (seen as negative values in Fig. S2(c), because locally $T_{\rm xx}^{20\ \rm MHz}$ becomes larger than $T_{\rm xx}^{10\ \rm MHz}$) and the formation of a stronger acoustic shadow behind the NA (seen as positive values in Fig. S2(c), because locally $T_{\rm xx}^{20\ \rm MHz}$ becomes smaller than $T_{\rm xx}^{10\ \rm MHz}$). The same behaviour is observed for the $T_{\rm yy}$ component and $T_{\rm xy}$ remains equal zero to machine accuracy, which clearly indicates (see the main text) that the quasi-static approximation is also valid at 20 MHz.

However, in a big picture, the difference between the stress components for the main harmonic and the second harmonic is negligibly small, which also makes it safe to assume that the difference between the first and the third harmonics will also be small. Consequently, at the frequencies of feasible harmonics the NA will behave similar to the case considered in much detail in the main text. From the optical point of view, the harmonics of ultrasound will produce the same patterns of dielectric permittivity variations as in the main text, but, according to the spectrum in Fig. S1(b), the magnitude of these variations will be lower. As a result, the intensity of the BLS peaks at the corresponding acoustic frequencies will also be lower.

It is noteworthy that the presented analysis allows avoiding computationally demanding simulations of the nonlinear acoustic interaction in the presence of the NA. In particular, we circumvent the scenario in which the nonlinear acoustic phenomena observable at the millimetre scale need to be modelled self-consistently with the acoustic scattering at the nano-scale. In this case, a 2 mm $\times$ 2 mm computational domain containing the NA would consists of $\sim 10^{12}$ discrete points. Because each point is used to store time-domain information about different acoustic and optical fields, this problem cannot be solved on supercomputers without additional simplifications.

Convergence of the Virieux-FDTD method

To verify the convergence of the Virieux-FDTD, we extended the simulation time up to 30 periods of oscillation and obtained the same result as for the three periods long simulations conducted in the main text. This unambiguously confirms that three periods long simulations produce converged results. All convergence tests were conducted using the 2D and 3D Virieux-FDTD methods [S9, S10], and the same convergence behaviour was observed for both 2D and 3D models. Consequently, below we present the result obtained using the 3D model only.

A workstation equipped with a quad-core Intel Xeon Processor and 16 GB 1600 MHz memory, running under Ubuntu 12.04 Linux operating system was used. The Simsonic software was obtained as a source code and compiled using OpenMP, an API for multi-platform shared-memory parallel programming in C/C++. The verification of the convergence of the 3D model took approximately two weeks of a continuous simulation run.

In Fig. S3 we show instantaneous snapshots of the $T_{\rm xx}$ amplitude profiles taken at the periods of time corresponding to different numbers of the oscillation cycle. One can see that the stress profile can be considered as converged after one oscillation period. Even when we run the simulation for just 0.05 period the resulting profile resembles the

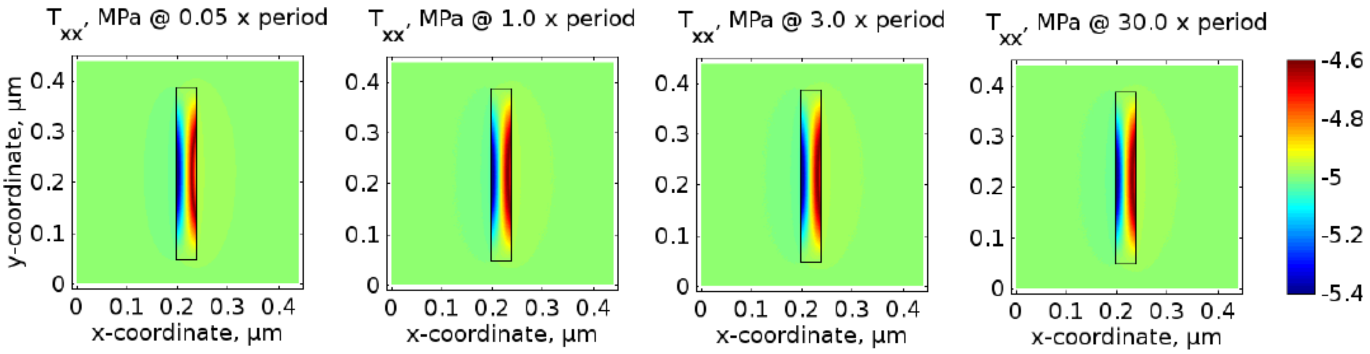

FIG. S3: Convergence behaviour of the amplitude of the stress component T_{xx} at the 10 MHz frequency of the incident acoustic wave. The number of oscillation periods is indicated in above the panels. The ultrasound propagates along the positive x-coordinate direction. All results were obtained using the 3D model.

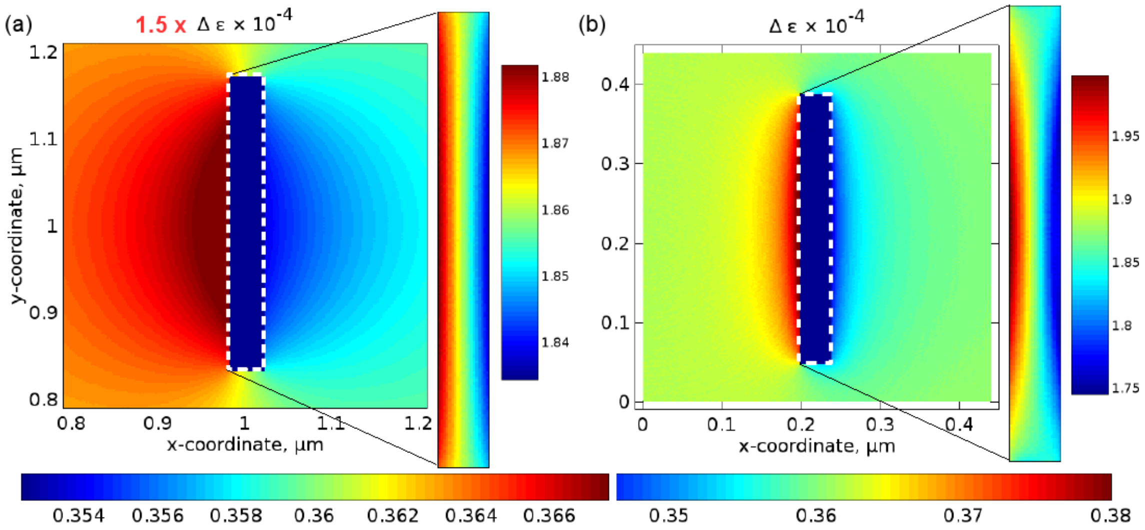

FIG. S4: The same results as in Fig. 4 in the main text, but in panel (a) $\Delta\epsilon$ is multiplied by 1.5, which is indicated above the panel. Note that this rescaling equalises the colour bar scales in the both panels, as can be seen, for example, by comparing the values encoded by the green colour.

converged result. A very similar convergence behaviour was reported in the previous works [S11, S12]. Finally, we note that the same convergence dynamics was observed in the case of the remaining components of the stress tensor.

Comparison of the dielectric permittivity perturbation profiles in the 2D and 3D models

In the main text, we demonstrated that the profile of the dielectric permittivity perturbations $\Delta\epsilon$ due to ultrasound produced by using the simplified 2D acoustic model is qualitatively similar to the profile obtained using the 3D acoustic model. However, in the 2D model the amplitude of $\Delta\epsilon$ of both silver and water is ~ 1.5 times smaller than in the 3D model. To demonstrate this difference, in Fig. S4 we plot the same result as in Fig. 4 in the main text, but in Fig. S4(a) we multiply the 2D-simulated amplitude of $\Delta\epsilon$ by 1.5. (The 3D-simulated result in Fig. S4(b) is the same as in Fig. 4(b).) One can see that after this rescaling the amplitudes of $\Delta\epsilon$ predicted by the 2D model and the 3D model become nearly the same. For example, in the colour bars of the main panels of Fig. S4(a, b) the green colour corresponds to $\Delta\epsilon \approx 0.36 \times 10^{-4}$ and in the insets the green colour corresponds to $\Delta\epsilon \approx 1.86 \times 10^{-4}$. We note that all colour bars are linear.

[S1] Hamilton, M. F. & Blackstock, D. T. *Nonlinear Acoustics.* (Academic Press, 1997).

[S2] Fabelinskii, I. L. *Molecular Scattering of Light.* (Springer, 1968).

[S3] Sebastian, T., Schultheiss, K., Obry, B., Hillebrands, B. & Schultheiss, H. Micro-focused Brillouin light scattering: imaging spin waves at the nanoscale. *Front. Phys.* **3,** 35 (2015).

[S4] Baudoin, M., Thomas, J.-L., Coulouvrat, F. & Chanéac, C. Scattering of ultrasonic shock waves in suspensions of silica nanoparticles. *J. Acoust. Soc. Am.* **129,** 1209–1220 (2011).

[S5] Gurbatov, S. N., Rudenko, O. V. & Hedberg, C. M. *Nonlinear Acoustic Through Problems ans Examples* (Trafford Publishing, 2009).

[S6] Sullivan, D. M. *Electromagnetic Simulation Using the FDTD Method* (IEEE Press, 2000).

[S7] Prieur, F. *Nonlinear Propagation of Ultrasonic Signals. Theoretical Studies and Potential Applications. PhD Thesis* (Faculty of Mathematics and Natural Sciences, University of Oslo, 2012).

[S8] Kaye, G. W. C. & Laby, T. H. *Tables of Physical and Chemical Constants and Some Mathematical Functions* (Longmans, 1959).

[S9] Bossy, E. *User's Guide for SimSonic2D.* (2012) Available at: http://www.simsonic.fr. (Accessed: 4th June 2016).

[S10] Bossy, E. *User's Guide for SimSonic3D.* (2012) Available at: http://www.simsonic.fr. (Accessed: 4th June 2016).

[S11] Zhao, H., Crozier, S. & Liu, F. A high definition, finite difference time domain method. *Appl. Math. Model.* **27,** 409–419 (2003).

[S12] Maksymov, I. S. & Kostylev, M. Microwave eddy-current shielding effect in metallic films and periodic nanostructures of sub-skin-depth thicknesses and its impact on stripline ferromagnetic resonance spectroscopy *J. Appl. Phys.* **116,** 173905 (2014).
